# Aerial Application of Mancozeb and Urinary Ethylene Thiourea (ETU) Concentrations among Pregnant Women in Costa Rica: The Infants’ Environmental Health Study (ISA)

**DOI:** 10.1289/ehp.1307679

**Published:** 2014-09-08

**Authors:** Berna van Wendel de Joode, Ana María Mora, Leonel Córdoba, Juan Camilo Cano, Rosario Quesada, Moosa Faniband, Catharina Wesseling, Clemens Ruepert, Mattias Öberg, Brenda Eskenazi, Donna Mergler, Christian H. Lindh

**Affiliations:** 1 Programa Infantes y Salud Ambiental (ISA), Central American Institute for Studies on Toxic Substances (IRET), Universidad Nacional, Heredia, Costa Rica; 2 Center for Environmental Research and Children’s Health (CERCH), School of Public Health, University of California at Berkeley, Berkeley, California, USA; 3 Division of Occupational and Environmental Medicine, Institute of Laboratory Medicine, Lund University, Lund, Sweden; 4 Institute of Environmental Medicine, Karolinska Institutet, Stockholm, Sweden; 5 Centre de recherche interdisciplinaire sur la biologie la santé et l’environnement (CINBIOSE), Université du Québec a Montréal, Montréal, Québec, Canada

## Abstract

Background: Mancozeb and its main metabolite ethylene thiourea (ETU) may alter thyroid function; thyroid hormones are essential for fetal brain development. In Costa Rica, mancozeb is aerially sprayed at large-scale banana plantations on a weekly basis.

Objectives: Our goals were to evaluate urinary ETU concentrations in pregnant women living near large-scale banana plantations, compare their estimated daily intake (EDI) with established reference doses (RfDs), and identify factors that predict their urinary ETU concentrations.

Methods: We enrolled 451 pregnant women from Matina County, Costa Rica, which has large-scale banana production. We visited 445 women up to three times during pregnancy to obtain urine samples (*n* = 872) and information on factors that possibly influence exposure. We determined urinary ETU concentrations using liquid chromatography mass spectrometry.

Results: Pregnant women’s median urinary ETU concentrations were more than five times higher than those reported for other general populations. Seventy-two percent of the women had EDIs above the RfD. Women who lived closest (1st quartile, < 48 m) to banana plantations on average had a 45% (95% CI: 23, 72%) higher urinary ETU compared with women who lived farthest away (4th quartile, ≥ 565 m). Compared with the other women, ETU was also higher in women who washed agricultural work clothes on the day before sampling (11%; 95% CI: 4.9, 17%), women who worked in agriculture during pregnancy (19%; 95% CI: 9.3, 29%), and immigrant women (6.2%; 95% CI: 1.0, 13%).

Conclusions: The pregnant women’s urinary ETU concentrations are of concern, and the principal source of exposure is likely to be aerial spraying of mancozeb. The factors predicting ETU provide insight into possibilities for exposure reduction.

Citation: van Wendel de Joode B, Mora AM, Córdoba L, Cano JC, Quesada R, Faniband M, Wesseling C, Ruepert C, Öberg M, Eskenazi B, Mergler D, Lindh CH. 2014. Aerial application of mancozeb and urinary ethylene thiourea (ETU) concentrations among pregnant women in Costa Rica: The Infants’ Environmental Health Study (ISA). Environ Health Perspect 122:1321–1328; http://dx.doi.org/10.1289/ehp.1307679

## Introduction

**Figure 1:**
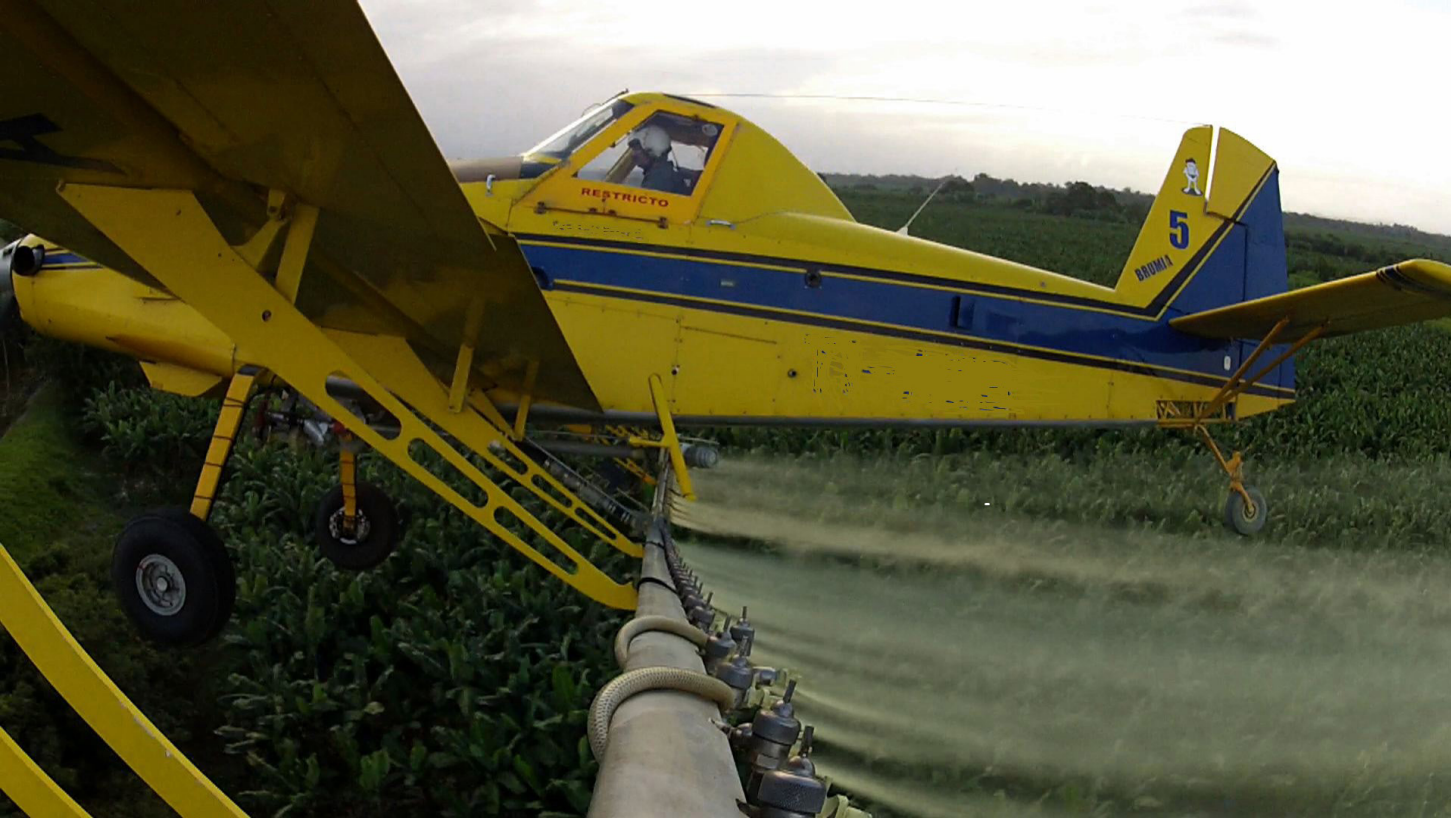
Aerial application of mancozeb at banana plantations in Costa Rica (photograph by Marcus Winterbauer,^©^ Längengrad Filmproduktion GmbH; reproduced with permission).

Banana export, primarily to the United States and Europe, is an important economic activity in Costa Rica, constituting 2.2% of the country’s gross domestic product and a source of employment for more than 40,000 workers [Corporación Bananera Nacional (CORBANA) 2012]. To protect banana plants from diseases such as black sigatoka, > 2 million kg of pesticides are applied annually on 40,000 hectares (Bravo et al. 2013; CORBANA 2012). The fungicide mancozeb, a manganese–zinc complex of ethylene-bis-dithiocarbamate (EBDC), comprises about half of the pesticides used and is applied weekly by light aircraft (Figure 1) (Barraza et al. 2011; Bravo et al. 2013). To our knowledge, no other EBDCs are being used on these plantations (Bravo et al. 2013). Mancozeb is a commonly used fungicide throughout the world, registered for use in almost 120 countries (Gullino et al. 2010). In the United States, approximately 3.4 million kg of mancozeb are applied annually in agriculture (National Toxicology Program 2014).

EBDCs are absorbed by skin, mucous membranes, and respiratory and gastrointestinal tracts, and metabolized via hepatic microsomal enzymes to produce ethylene thiourea (ETU) (Houeto et al. 1995). ETU is rapidly absorbed via the gastrointestinal tract, subsequently filtered by kidneys, and excreted in urine (World Health Organization 1988). ETU is also present as a 0.01–4.5% impurity in EBDC formulations (Camoni et al. 1988; Lindh et al. 2008).

Although both mancozeb and ETU possess low acute toxicity [U.S. Environmental Protection Agency (EPA) 2005], neuroblastic necrosis and hydrocephaly have been reported in ETU-exposed rat embryos at doses far lower than those that caused observable toxic signs in the rat dam (Khera 1987). ETU also is a known inhibitor of thyroid peroxidase activity and alterations in thyroid weight, cells, hormones, and iodine uptake, and thyroid tumors have been reported in chronic mancozeb- and ETU-exposed rats, mice, and dogs (Axelstad et al. 2011; Belpoggi et al. 2002; Chhabra et al. 1992; International Agency for Research on Cancer 2001).

Two cross-sectional studies of EBDC-exposed Mexican backpack applicators (*n* = 49) (Steenland et al. 1997) and Philippine banana plantation workers (*n* = 57) (Panganiban et al. 2004) have reported higher mean serum thyroid-stimulating hormone (TSH) concentrations compared to nonexposed workers, although the difference was not statistically significant for the Philippine banana workers. In addition, the Mexican sprayers had higher mean sister chromatic exchanges and chromosome translocations than those who were nonexposed (*n* = 31), suggesting that cytogenic effects may be associated with EBDC exposure (Steenland et al. 1997). Panganiban et al. (2004) reported a positive correlation between ETU concentrations measured in blood and size of solitary thyroid nodules measured with thyroid gland ultrasounds. Possible effects of mancozeb and ETU on thyroid function are of particular concern for fetal brain development, which requires adequate thyroid hormone secretion: Even mild maternal alterations may affect fetal neurological development (Kester et al. 2004; Patel et al. 2011).

Urinary ETU concentrations are considered a well-established biomarker to evaluate mancozeb and ETU exposures from occupation, environment, and diet (Lindh et al. 2008). Median urinary ETU concentrations in EDBC-exposed workers from vineyards, greenhouses, and potato farms ranged from 2 to 45 μg per gram creatinine (g.cr) (Colosio et al. 2002; Fustinoni et al. 2005, 2008; Kurttio and Savolainen 1990; Sottani et al. 2003). Median urinary ETU concentrations in general populations from Italy, the United Kingdom, and the United States are generally below the limit of detection (LOD) (< 0.5 μg/g.cr) (Aprea et al. 1996; Castorina et al. 2010; Colosio et al. 2006; Jones et al. 2010; Saieva et al. 2004). Detectable urinary ETU concentrations in general populations are thought to be attributable to exposure to EBDCs and ETU from consumption of foods with pesticide residues (Aprea et al. 1997).

Reverse dosimetry allows interpretation of urinary biomarkers through comparisons with reference doses (RfDs) (Clewell et al. 2008). An RfD is an estimate of daily exposure to the human population that is thought to be without an appreciable risk of deleterious effects during a lifetime (U.S. EPA 1996). The U.S. EPA Integrated Risk Information System (IRIS) has set the RfD for chronic oral ETU exposure at 0.08 μg/kg/day (U.S. EPA 1996). This RfD was derived from a lowest observed effect level (LOAEL) of 0.25 mg/kg/day for thyroid hyperplasia in rats (Graham et al. 1975) and includes a total uncertainty factor of 3,000 to account for inter- and intraspecies differences (100*×*), limited developmental toxicological and multigeneration data (3×), and observed effects at lowest dose tested (10×). The U.S. EPA Office of Prevention, Pesticides and Toxic Substances (OPPTS) has set an alternative RfD—the chronic population-adjusted dose (cPAD)—at 0.18 μg/kg/day (U.S. EPA 2005). This cPAD is based on a no observed effect level (NOAEL) of 0.18 mg/kg/day for thyroid toxicity in dogs and includes a total uncertainty factor of 1,000: 100× for inter- and intraspecies variation and 10× for lack of data. The U.S. EPA (2005) has also set a PAD for acute exposure (aPAD) at 5 μg/kg/day. This aPAD is based on a NOAEL of cerebellum Purkinje cell migration in rat embryos after maternal exposure to 5 mg ETU/kg/day (Khera 1973) and includes a total uncertainty factor of 1,000: 100× for inter- and intraspecies variation, 10× for lack of data on developmental neurotoxicity studies.

To our knowledge, only one previous study (CHAMACOS; Center for the Health Assessment of Mothers and Children of Salinas) has measured urinary ETU concentrations in pregnant women who lived in an agricultural area with ground spraying of mancozeb and maneb and whose urine ETU concentrations were below the LOD of 0.1 μg/L (Castorina et al. 2010). In developing countries, where extensive pesticide application methods such as aerial spraying are common, information is lacking. Because the fetus and newborn may be at particular risk for health effects from mancozeb and ETU exposure, we performed a study to *a*) evaluate urinary ETU concentrations in pregnant women living near large-scale banana plantations with extensive mancozeb use, *b*) assess whether pregnant women’s estimated daily intake exceeds established RfDs, and *c*) identify factors that predict their urinary ETU concentrations.

## Material and Methods

**Figure 2:**
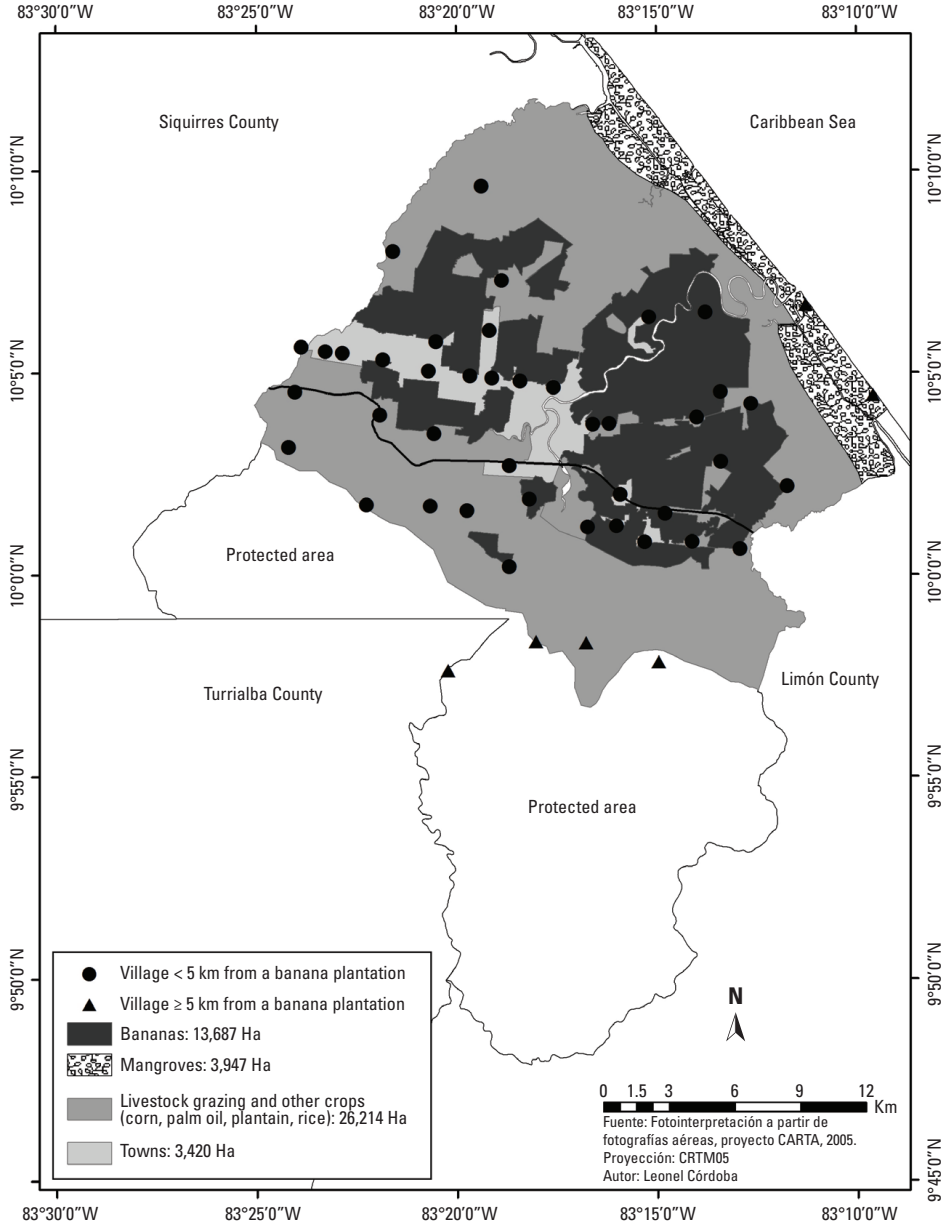
Land use in Matina County where large-scale banana plantations constitute the main economic activity.

*Study population*. The Infants’ Environmental Health Study [Infantes y Salud Ambiental (ISA)] is a prospective community-based birth cohort study in Matina County, Limón, Costa Rica, aimed at examining possible effects of prenatal pesticide and manganese exposure on children’s growth and neurodevelopment. The population of Matina County is approximately 37,700 [Costa Rican Institute of Statistics and Census (INEC) 2011], and large-scale banana plantations constitute the main economic activity, representing 34% of the area used for agriculture and livestock grazing (Figure 2). On these plantations, mancozeb is applied weekly by light aircraft.

Pregnant women were eligible for enrollment if they lived in one of 40 villages in Matina County that were within 5 km of a banana plantation, if they were at least 15 years old, with < 33 weeks of gestation, and if they expected a singleton birth. Women were identified between March 2010 and June 2011 through meetings in local schools, communal groups, advertisements, and referral.

A total of 480 eligible pregnant women were identified; of these, 451 (94%) agreed to participate. Written informed consent was obtained from each woman. For women < 18 years of age, additional written informed consent was obtained from their legal representative. The women did not receive any incentives for their participation. All study activities were approved by the Scientific Ethics Committee of the Universidad Nacional in Costa Rica (CECUNA-11-2009).

*Study procedure*. Women were interviewed in their homes one to three times during pregnancy depending on gestational age at enrollment. Mean (± SD) time between first and second, and second and third, visit was 10.6 ± 4.0 and 9.6 ± 3.6 weeks, respectively. A urine sample was obtained at each visit. During the visits blood and hair samples were also collected; results from these samples have been reported elsewhere (Mora et al. 2014). In addition, clinical information was abstracted from the Prenatal Health Card, which is provided to pregnant women by the Costa Rica Social Security (CCSS) and is completed by physicians and nurses at each prenatal care visit. Gestational age at day of visit was calculated on the basis of the first day of the last menstrual period (LMP) as reported by each woman. When LMP was unknown (*n* = 18), gestational age was based on ultrasound (*n* = 5) or physicians report (fundal height, *n* = 10). Three women lacked information on gestational age and were excluded from data analysis. An additional three women did not provide a urine sample. We obtained 872 urine samples from the remaining 445 women. Mean gestational age at the first, second, and third urine collection was 18.3 ± 6.5 (*n* = 440), 28.7 ± 5.9 (*n* = 330), and 32.9 ± 2.7 (*n* = 102) weeks, respectively.

*Interview*. At each visit, interviewers used structured questionnaires to obtain information on women’s sociodemographic characteristics, medical and occupational history, partners’ occupation, pesticide use at work and at home, lifestyle habits, quality and type of housing, basic dietary information (including frequency of fruit and vegetable intake), and source of drinking water. For comparison, our questionnaires were based on instruments used in the CHAMACOS study (Eskenazi et al. 2003) and adapted to locally used Spanish. We also asked women whether they had washed agricultural work clothes on the day before sampling and the day of sampling, about nearby aerial applications on the day before and day of sampling, and about residential agrochemical use on the day before and day of urine collection.

*Georeferencing*. We documented participants’ residential coordinates, using a global positioning system (GPS) receiver (Garmin Etrex Venture HCto). Coordinates were located on a geocoded map of Matina County, using ArcGIS 10.0 software (ESRI, Redlands, CA, USA). We also recorded GPS coordinates of banana plantations within a 5-km radius of each home, using aerial photographs [CARTA (Costa Rica Airborne Research and Technology Applications) project 2005; Centro Nactional de Alta Tecnología 2011]. Plantations were measured as static areas of at least four points when possible. Euclidean distances were measured from residence to the nearest border of closest banana plantation.

*Urinary sampling and ETU analysis*. Urine samples were collected in 100-mL beakers (Vacuette®, sterile), transferred to 15 mL tubes (PerformR™ Centrifuge tubes, Labcon®, sterile), and stored at –20°C until shipment (4°C) to Lund University, Sweden, for analysis. Samples were hydrolyzed in 0.09 M NaOH for 1 hr at 100°C and aliquots of 20 μL were analyzed using a triple quadrupole linear ion trap mass spectrometer (QTRAP 5500; AB Sciex, Foster City, CA, USA) coupled to a two dimensional liquid chromatography system (UFLCRX; Shimadzu Corporation, Kyoto, Japan) (Ekman et al. 2013). The analyses were performed in positive atmospheric pressure chemical ionization mode (Ekman et al. 2013). Three urinary quality control samples with known concentrations were added to each analytical batch (*n* = 28). The coefficients of variation (CVs) were 12, 8, and 6%, for 2.5, 7.6, and 32.7 μg ETU/L, respectively. The LOD was 0.1 μg ETU/L urine, estimated by injecting urine samples with known low ETU concentrations and calculated by mean peak level within 0.1 min of expected retention time of ETU, plus three times the standard deviation of the peak (Miller and Miller 2005). All samples were prepared in duplicates, worked up, and analyzed on different days. A between-batch precision was determined from 300 duplicate quantified values with CVs of 12, 9, and 8% at 1.0 (*n* = 100), 2.3 (*n* = 100), and 8.5 (*n* = 100) μg ETU/L, respectively. Average urinary ETU concentration was calculated for each duplicate and used in statistical analysis. We also determined urinary density (hand refractometer) (kilograms per liter) and creatinine concentrations (grams creatinine per liter) (Mazzachi et al. 2000). We calculated specific gravity–corrected ETU concentrations (ETU-sg) expressed as micrograms ETU/L_specific gravity-corrected_ urine as well as creatinine-corrected ETU concentrations (ETU-cr) expressed as micrograms ETU/gram creatinine.

*EDIs*. For comparison with chronic RfDs, we estimated each woman’s daily ETU intake (micrograms ETU/kilograms body weight per day), by reverse dosimetry from their average urinary ETU-cr, using a one-compartment first-order kinetic model and assuming steady state (Clewell et al. 2008):

*EDI_chronic_* = *ETU-cr* × *BW*^–1^ × *C* × (*ln*2/*t*½) × *AR*^–1^ × *E*^–1^, [1]

where *ETU-cr* is the average woman’s creatinine-corrected ETU concentration (micrograms per gram creatinine), *BW* is her body weight before pregnancy (kilograms), *C* is the estimated daily creatinine release in pregnant women (1.21 g) (Chattaway et al. 1969), *t*½ is estimated half-life of ETU (0.83 days) (Lindh et al. 2008), *AR* is the estimated gastrointestinal absorption rate (50%) (European Commission 2009), and *E* is the urinary excretion rate for ETU [90%, based on studies in rhesus monkeys and rats by Allen et al. (1978)].

In addition, for comparison of women’s EDIs with the aPAD, we also estimated EDI_acute_ from each urinary ETU-cr separately (*n* = 872).

*Statistical analysis*. We used descriptive statistics to examine the relationships of general, occupational, and environmental characteristics. We tested whether continuous variables followed a normal distribution (Shapiro–Wilk *W*-test). We compared associations between categorical variables with Pearson’s chi-square test test for categorical measures. We determined associations between categorical and continuous variables with Student’s *t*-test for continuous normally distributed measures (education), and Wilcoxon/Kruskal–Wallis rank sum test for continuous measures that were not normally distributed (age, income per capita, distance, and urinary ETU concentration). Correlations between continuous variables were estimated using Spearman’s *r* correlation coefficient. All urine samples had ETU concentrations above the LOD. Because urinary ETU concentrations followed a lognormal distribution, natural logarithmic-transformed ETU concentrations were used in statistical models. One urine sample with an extremely high ETU concentration of 207 μg/L was excluded from statistical analysis.

We used variance components models with random intercepts for each participant to estimate between- and within-woman variability, and intraclass correlation coefficients (ICCs) of lnETU, lnETU-cr, and lnETU-sg concentrations from repeated samples collected from the same woman. ICCs are often used to estimate temporal reliability of biomarkers (Rosner 2006). We examined possible differences in lnETU, lnETU-cr, and lnETU-sg between trimesters using mixed-effects regression models with random intercepts for each participant and including trimester as an independent variable. In addition, we used paired *t*-test of lnETU concentrations to compare mean differences between first, second, and third urine samples.

To identify factors that predicted urinary lnETU concentrations (*p* < 0.1) and were reported by at least 5% of the women, we used linear mixed-effects regression models with random intercepts, taking into account correlation among repeat samples collected from the same woman (Peretz et al. 2002). To correct for urinary dilution, we included creatinine concentration (micrograms per liter) as an independent variable in all models. The latter allows proper adjustment of urinary ETU for creatinine, while assuring the statistical significance of other variables in the model to be independent of effects of urinary concentration (Barr et al. 2005). First, we ran bivariate models of the following factors: gestational age at sampling (weeks), woman’s age (years at enrollment), woman’s and partner’s educational level (completed years of education), marital status (married or living as married/single), family income per capita (US$ per capita), country of birth (Costa Rica/other Central American countries), residential distance to banana plantation (meters, grouped into quartiles), work in agriculture during pregnancy (yes/no), partner’s occupation in agriculture (yes/no), living with agricultural workers (yes/no), washing agricultural work clothes on the day before sampling and the day of sampling (yes/no, as separate variables), nearby aerial applications on the day before and day of sampling (yes/no, as separate variables), pesticide use inside or around the home on the day before and day of sampling (yes/no, as separate variables), source of drinking water [aqueduct/other (well, rain water, or river)], and dietary variables such as maternal consumption of fruits (< 5 or ≥ 5 times/week), consumption of green bananas and plantains (< 10 or ≥ 10 times/week), vegetables (< 5, ≥ 5–10, or ≥ 10 times/week), and rice and beans (< 15 or ≥ 15 times/week). We included all factors with *p* < 0.2 in a multivariable linear mixed-effects model and used manual stepwise selection to retain factors with *p* < 0.1 in the final multivariable model.

For categorical variables we expressed estimated coefficients as the percent difference from the mean of the reference category by exp(β) (exponentiated regression coefficients) (Kennedy 1981). For example, for women who worked in agriculture during pregnancy the reference condition was women who did not work in agriculture during pregnancy. For continuous variables, we calculated percent difference in urinary ETU concentration associated with a 1-unit increase in the independent variable while all other variables in the model are held constant, by [exp(β) – 1] × 100 (Halvorsen and Palmquist 1980). To test for a trend between residential distance to banana plantation and urinary ETU, we also added distance (meters) to the model as a continuous variable after natural log-transformation. This log-transformation was done to ensure homoscedasticity of residuals. The estimated β represents the percent change in *y* (dependent variable) while *x* (independent variable) increases by 1% (Institute for Digital Research and Education 2014). Residuals of the regression models were tested for normality (Shapiro–Wilk *W*-test) and outliers. To assess influence of outliers on regressions, we performed additional analyses excluding the 1% of observations with the highest Cook’s distance values (Zuurbier et al. 2011). For all statistical tests, the significance level was set at 5%. We used JMP 8 (SAS Institute Inc., Cary, NC, USA) for statistical analysis.

## Results

In general, women were young, with 25% who were ≤ 19 years at enrollment, and economically impoverished with a median income below the Costa Rica poverty line of US$142 per capita per month (INEC 2012) (Table 1). Twenty five percent of the women lived within 50 m of a banana plantation (Table 1). At enrollment, 7% of the women and 57% of their partners worked at banana plantations, and 1% of the women and 6% of their partners performed other agricultural work (Table 2). None of the women reported applying mancozeb herself at work. Also, none of the women reported residential use of mancozeb or other EBDCs. Nineteen percent of the women were immigrants, almost exclusively from Nicaragua (Table 2). Women frequently consumed rice and beans, 30% more than 15 times/week (Table 2).

**Table 1:** Description of pregnant women from the ISA birth cohort study with at least one urine sample (*n* = 445).

Characteristic	*n*	Mean ± SD	P50 (P25, P75)	Minimum	Maximum
Age at enrollment (years)	445	24 ± 6.5	22 (19, 28)	15	44
Gestational age at enrollment (weeks)	445	18 ± 6.4	18 (13, 24)	6	33
Educational level (completed years)	445	7.0 ± 2.8	6.0 (6.0, 9.0)	0	15
Income per capita (US$/month)	412	140 ± 93	120 (80, 173)	16	1,080
Partner’s age (years)	438	28 ± 9.0	26 (22, 33)	15	64
Partner’s educational level (completed years)	391	6.6 ± 2.9	6.0 (6.0, 9.0)	0	16
Residential distance to banana plantation at enrollment (m)	445	453 ± 657	216 (48, 565)	0.3	4,115
P, percentile.					

**Table 2:** General, occupational, environmental, and dietary characteristics of pregnant women from the ISA birth cohort study with at least one urine sample (*n* = 445).

Characteristic	*n* (%)
Marital status
Married/living as married	336 (76)
Single	109 (24)
Country of birth
Costa Rica	361 (81)
Other Central American^*a*^	84 (19)
Smoking during pregnancy
Yes	18 (4)
No	426 (96)
≥ 1 glass of alcohol consumption during pregnancy
Yes	14 (3)
No	428 (97)
Drug use during pregnancy
Yes	5 (1)
No	438 (99)
Source of drinking water
Aqueduct	348 (78)
Other: well, rain water, river	97 (22)
Occupation at enrollment
Working in banana plantations	33 (7)
Other agricultural work	3 (1)
Other work (not agricultural)	78 (16)
Housewives and/or not working	331 (76)
Partner’s occupation at enrollment
Working on banana plantations	245 (57)
Other agricultural work	25 (6)
Other work (not agricultural)	132 (31)
Not working	25 (6)
Living with agricultural worker(s) during pregnancy
Yes	347 (78)
No	98 (22)
Washed agricultural work clothes on day of sample collection
Yes	87 (20)
No	345 (80)
Washed agricultural work clothes on day before sample collection
Yes	84 (20)
No	341 (80)
Aerial spraying near residence on day of sample collection
Yes	106 (25)
No	323 (75)
Aerial spraying near residence on day before sample collection
Yes	104 (25)
No	311 (75)
Consumption of green bananas or plantains
< 5 times/week	167 (38)
≥ 5 but < 10 times/week	169 (39)
≥ 10 times/week	99 (23)
Consumption of other vegetables
< 5 times/week	89 (20)
≥ 5 but < 10 times/week	126 (29)
≥ 10 but < 15 and times/week	192 (44)
≥ 15 times a week	31 (7)
Consumption of rice and beans
< 10 times/week	168 (38)
≥ 10 but < 15 times/week	139 (32)
≥ 15 times/week	134 (30)
Consumption of fruits
< 1 times/week	61 (14)
1–2 times/week	181 (41)
≥ 3 but < 5 times/week	95 (22)
≥ 5 times/week	103 (23)
Information was missing for several women with at least one urine sample: smoking (*n* = 1), alcohol use (*n* = 3), drug use (*n* = 2), partner’s occupation (*n* = 18), washing work clothes day of (*n* = 13)/before (*n* = 20) sampling, aerial spraying day of (*n* = 16)/before (*n* = 20) sampling, consumption of vegetables (*n* = 7), consumption of green bananas/plantains (*n* = 10), consumption of rice and beans (*n* = 4), consumption of fruits (*n* = 5). ^***a***^All immigrant women were born in Nicaragua except for one who was born in El Salvador.	

Pregnant women’s ETU, ETU-sg, and ETU-cr concentrations were similarly distributed, with median concentration of 2.9 μg/L [interquartile range (IQR) = 1.8–4.6], 3.1 μg/L_sg_ (IQR = 2.0–4.5), and 3.0 μg/g.cr (IQR = 1.9–4.6), respectively (Table 3). There were no significant differences in geometric mean concentrations according to the trimester in which samples were collected (Table 3). For example, compared with samples collected in the first trimester, geometric mean ETU-cr concentrations were 3.8% (95% CI: –12, 22) and 4.7% (95% CI: –11, 24) higher for samples collected in the second and trimesters, respectively (data not shown). When restricting analysis to women with three repeat samples (*n* = 90), we also did not detect significant differences in geometric mean ETU concentration between trimesters, or between first, second, or third urine samples. For example, compared with first sample, differences were –0.6% (95% CI: –17, 20) and –5.0% (95% CI: –22, 16) for second and third sample, respectively. Concentrations varied more within women than between women, which was reflected by the relatively low overall ICCs of 15% to 19% (Table 3).

**Table 3:** Distribution and variability of urinary ETU concentrations from pregnant women from the ISA birth cohort study.

ETU	No. of samples (no. of women)	Mean ± SD	GM (GSD)	Minimum	P10	P25	P50	P75	P90	Maximum	S^2^_B_^*a*^	S^2^_W_	ICC
Uncorrected (μg/L)
Overall	872 (445)	4.2 ± 8.0	2.9 (2.2)	0.3	1.1	1.8	2.9	4.6	7.5	207.0	1.12	1.63	0.18
1st trimester^*b*^	118 (117)	4.6 ± 6.3	3.1 (2.2)	0.5	1.3	1.9	2.6	4.7	8.2	42.0	—	—	—
2nd trimester	404 (367)	3.6 ± 2.9	2.8 (2.1)	0.3	1.1	1.8	2.9	4.4	6.9	23.9	1.05	1.59	0.10
3rd trimester	350 (306)	4.7 ± 11.7	2.9 (2.4)	0.3	1.0	1.7	3.1	4.7	8.4	207.0	1.23	1.60	0.31
Corrected (μg/L_specific gravity-corrected_)
Overall	872 (445)	4.1 (8.6)	3.1 (1.9)	0.2	1.5	2.0	3.0	4.5	6.8	236.3	1.06	1.40	0.15
1st trimester^*c*^	118 (117)	4.1 (4.2)	3.1 (2.0)	0.6	1.3	2.0	2.7	4.9	6.8	29.2	—	—	—
2nd trimester	404 (367)	3.6 (2.5)	3.0 (1.8)	0.2	1.5	2.0	2.9	4.3	6.3	20.1	1.10	1.26	0.28
3rd trimester	350 (306)	4.7 (13.0)	3.2 (1.9)	0.5	1.4	2.0	3.1	4.7	7.3	236.3	1.16	1.35	0.33
Corrected (μg/g creatinine)
Overall	870 (445)	4.1 ± 7.5	3.0 (2.0)	0.1	1.3	1.9	2.9	4.5	6.9	196.6	1.09	1.46	0.19
1st trimester^*d*^	117 (117)	3.9 ± 3.7	2.8 (2.1)	0.6	1.1	1.7	2.8	4.8	7.4	20.4	—	—	—
2nd trimester	404 (367)	3.8 ± 3.0	3.0 (2.0)	0.1	1.4	1.9	2.9	4.5	6.8	21.1	1.14	1.32	0.32
3rd trimester	349 (306)	4.6 ± 11.1	3.1 (2.1)	0.4	1.3	2.0	3.0	4.6	6.9	196.6	1.16	1.42	0.30
Abbreviations: GM, geometric mean; GSD, geometric standard deviation; P, percentile; S^2^_B_, variance between women; S^2^_W_, variance within women. ^***a***^The sample with the maximum ETU concentration (207 μg/L, 236.3 μg/Lsg, and 196.6 μg/g.cr) was excluded from analysis for estimation of variance components, ICC, and estimation of percent difference between trimesters of pregnancy. ^***b***^Percent difference in ETU was –13% (95% CI: –35, 6.3%) and –8.0% (95% CI: –0.30, 11%) for second and third trimester compared with first trimester, respectively. ^***c***^Percent difference in ETU-sg was –4.5% (95% CI: –22, 10%) and –4.1% (95% CI: –16, 6.2%) for second and third trimester compared with first trimester, respectively. ^***d***^Percent difference in ETU-cr was 3.8% (95% CI: –12, 22%) and 4.7% (95% CI: –11, 24%) for second and third trimester compared with first trimester, respectively.													

**Figure 3:**
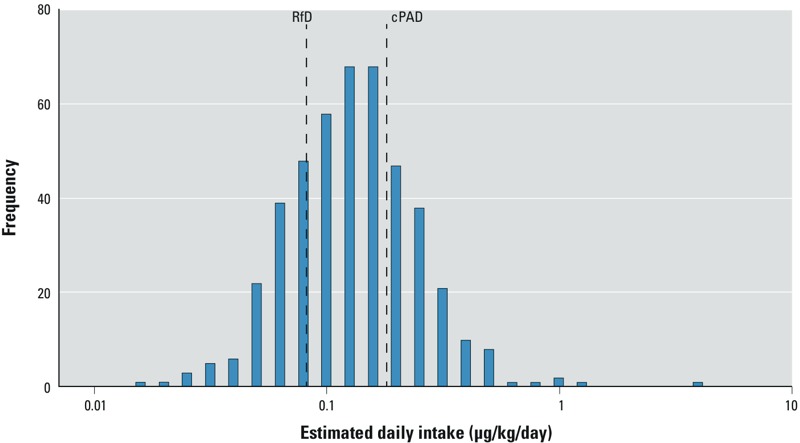
Histogram of the estimated daily intake (log-scale), expressed as μg ETU/kg body weight per day, in pregnant women from the ISA study, in relation to the chronic reference doses of 0.08 μg/kg per day (U.S. EPA 1996) and 0.18 μg/kg per day (U.S. EPA 2005).

Median EDI_chronic_, estimated from each woman’s average urinary ETU-cr, was 0.12 μg ETU/kg/day (IQR = 0.08–0.17 μg/kg/day) (Figure 3). Variability in EDI_chronic_ was relatively small: a factor of 2.9 between the 95th and 50th percentile. More than 72% of the women had an EDI_chronic_ above the RfD of 0.08 μg/kg/day (U.S. EPA 1996), and 23% of the women had an EDI that was also above the cPAD of 0.18 μg/kg/day (U.S. EPA 2005). The 95th percentile of the EDI_chronic_ distribution (0.33 μg/kg/day) was more than four times the RfD, and twice the cPAD. For acute exposure, there was only one very high EDI_acute_ (7.71 μg/kg/day), which exceeded the aPAD of 5 μg/kg/day (U.S. EPA 2005). The woman with this high value had a urinary ETU concentration of 196.6 μg ETU/g.cr on one of her measurement days. A second urine sample obtained from the same woman 6 weeks later was much lower (2.6 μg ETU/gr.cr). The woman lived 10 m from a banana plantation and mentioned in an open question her concern about living near aerial spraying. Apart from living near banana plantations, she did not report additional factors that could explain this high ETU-cr. The 95th percentile of EDI_acute_ was 0.37 μg/kg/day, more than 10 times lower than the aPAD.

In Table 4 we present results of the bi- and multivariate mixed-effects models of factors associated with urinary ETU concentration. Results from bivariate analysis showed that women who lived closer to banana plantations had higher ETU concentrations than did women who lived at a larger distance (Table 4). Women who worked in agriculture during pregnancy and women who washed work clothes on the day of or day before sampling likewise had higher ETU than women who did not (Table 4). The association was stronger for washing work clothes the day before sampling compared with washing work clothes on the day of sampling (Table 4). Women who reported near aerial spraying on the day of sampling also had higher ETU concentrations (Table 4), but no significant difference was detected for spraying reported on the day before sampling (1.1% higher; 95% CI: –4.2, 7.0, *p* = 0.69). Immigrant women had higher ETU compared with Costa Rican–born women (Table 4). Women who reported eating rice and beans ≥ 15 times had higher ETU concentrations than did women who consumed rice and beans less frequently (Table 4), but no statistically significant differences were detected for the other dietary factors (data not shown). Also, no statistically significant differences were observed for age, women’s or partners’ educational level, marital status, income per capita, partner’s occupation, living with agricultural workers, source of drinking water, and residential pesticide use on the day of/before sampling (data not shown).

**Table 4:** Results of bi- and multivariate mixed effect models of factors associated (*p* < 0.1) with pregnant women’s urinary ETU concentrations (*n* = 833 samples, 437 women), all models included creatinine as independent co-variable.^*a*^

Factor	Bivariate % difference (95% CI)	*p*-Value	Multivariate % difference (95% CI)	*p*-Value
Creatinine (μ/L)	77 (66, 89)	< 0.0001	80 (69, 92)	< 0.0001
Residential distance to banana plantation (m)^*b,c*^
< 48 (1st quartile)	52 (28, 80)	< 0.0001	45 (23, 72)	< 0.0001
≥ 48–216 (2nd quartile)	18 (–1.0, 40)	0.07	12 (–5.4, 38)	0.31
≥ 216–565 (3rd quartile)	17 (–1.1, 39)	0.07	17 (–1.2, 38)	0.08
≥ 565 (4th quartile)	—	—	—	—
Occupation in agriculture during pregnancy	19 (9.0, 30)	< 0.001	19 (9.3, 29)	< 0.0001
Washed agricultural work clothes on day of sampling	5.8 (–0.2, 12)	0.06
Washed agricultural work clothes on day before sampling	13 (6.2, 19)	< 0.0001	11 (4.9, 17)	0.0003
Near aerial spraying day of sample collection	7.2 (1.4, 13)	0.01
Consumption of rice and beans ≥ 15 times a week	5.1 (–0.3, 11)	0.07
Immigrant	10 (3.4, 17)	0.003	6.2 (1.0, 13)	0.049
^***a***^Information was missing for several women with at least one urine sample for washed agricultural work clothes on day of sample collection (*n* = 10 samples, 3 women), for near aerial spraying on day of sampling (*n* = 12 samples, 3 women), and for consumption of rice and beans (*n* = 5 samples, 3 women). ^***b***^When grouping 2nd and 3rd quartile together percent difference was 18% (95% CI: 2.7, 35%; *p* = 0.01) for bivariate model and 14% (95% CI: 0.3, 30%, *p* = 0.04) for multivariate model, respectively. ^***c***^When we included distance as a continuous variable (ln-meters) instead of categorical variable, we detected a negative association between residential proximity to banana plantation (ln-meters) and lnETU; for bivariate model: β = –0.09 (95% CI: –0.12, –0.06) (*p* < 0.0001), for multivariate model: β = –0.08 (95% CI: –0.11, –0.05) (*p* < 0.0001).				

In the multivariate model, residential proximity to banana plantation, maternal occupation in agriculture, washing agricultural work clothes on the day before visit, and being an immigrant were retained (all *p* < 0.05). The adjusted differences in urinary ETU for the factors in the multivariate model were somewhat smaller compared with the difference from the bivariate models (Table 4).

Results from multivariate analysis showed that women who lived closer to banana plantations had higher ETU concentrations than women who lived at a larger distance (Table 4). Indeed, those who lived within 48 m from a plantation (1st quartile) had 45% (95% CI: 23, 72%) higher urinary ETU concentrations compared with women who lived ≥ 565 m (4th quartile) (Table 4). We detected a negative trend between residential proximity to banana plantation (ln-meters) and lnETU concentrations: β = –0.08 (95% CI: –0.11, –0.05) (*p* < 0.0001) (see Supplemental Material, Figure S1). Women who worked in agriculture during pregnancy had a 19% (95% CI: 9.3, 29%) higher ETU concentrations compared with women who did not (*p* < 0.001). Women who washed work clothes on the day before sampling likewise had higher ETU concentrations than other women (Table 4) (11% higher; 95% CI: 4.9, 17%) (*p* < 0.0003). On average, immigrant women had 6.2% (95% CI: 1.0, 13%) higher ETU concentrations than Costa Rican–born women (*p* = 0.049). Because the immigrant women may have different environmental and social characteristics compared with Costa Rican–born women, we analyzed sociodemographic, environmental, and occupational characteristics of Costa Rican–born (*n* = 361) versus immigrants (*n* = 84). Characteristics generally were similar for both groups, but immigrants lived closer to the banana plantations (median distance 98 m (IQR = 19, 366) versus 267 m (IQR = 75, 602) (*p* < 0.001) compared with Costa Rican–born women, and more immigrant women washed agricultural work clothes on the day before sampling compared with Costa Rican–born women, 39% versus 15%, respectively (*p* < 0.0001) (Table 5).

**Table 5:** Sociodemographic, environmental, and occupational characteristics of Costa Rican–born (*n* = 361) and immigrant (*n* = 84) women.

Characteristic	Costa Rican born (*n* = 361)	Immigrants (*n* = 84)	*p*-Value
Age (years) [median (IQR)]	22.1 (18.8, 27.3)	23.9 (20.2, 30.1)	0.05
Education (mean ± SD)	7.1 ± 2.6	6.5 ± 3.2	0.08
Distance to banana plantation (m) [median (IQR)]	98 (19, 366)	267 (75, 602)	0.0007
Income/per capita (US$) [median (IQR)]	120 (77, 172)	120 (80, 200)	0.55
Work in agriculture (%)	7.5%	10.7%	0.33
Near aerial spraying
Day of sampling	23%	33%	0.08
Day before sampling	26%	23%	0.60
Washing agricultural work clothes
Day of sampling	18%	28%	0.05
Day before sampling	15%	39%	< 0.0001

## Discussion

The results of this study show elevated urinary ETU in pregnant women living in the vicinity of banana plantations. Urinary ETU was associated with residential proximity to a banana plantation, washing agricultural work clothes on the day before sampling, and working in agriculture during pregnancy. Immigrant women had higher urinary ETU, but this was explained partly by environmental and social factors because, compared with the Costa Rican–born women, they lived closer to the plantations, and proportionally more washed agricultural clothes the day before sampling.

Frequent mancozeb spraying by light aircraft over the banana plantations is the probable source of these elevated concentrations of urinary ETU and their variation with environmental and occupational factors. To our knowledge, aerial spraying is the only way in which mancozeb is applied on Costa Rican large-scale banana plantations; in this study, mancozeb was not reported being used for residential purposes. Because only 1% of the women and only 6% of their partners performed other agricultural work, the elevated urinary ETU concentrations in pregnant women who worked in agriculture and washed agricultural work clothes are likely to be a consequence of aerial spraying activities of mancozeb at banana plantations. Women working at banana plantations in general are employed in the packing plants that are located inside the banana plantations and can easily be contaminated when bananas are aerially sprayed. None of the women from this study reported applying mancozeb herself.

The agricultural work clothes the women most often washed came from family members who worked on banana plantations. In this activity, there was a stronger association of ETU with washing the day before urine sampling compared with washing on the day of urine sampling, which may reflect relatively slow uptake of ETU after dermal exposure (Ekman et al. 2013). In contrast, ETU was more strongly associated with reported aerial spraying on the day of sampling than aerial spraying on the previous day, possibly due to faster absorption of mancozeb and ETU through the lungs following respiratory exposure compared with absorption via dermal exposure (Ekman et al. 2013).

These findings suggest that both respiratory and dermal exposure may be relevant routes of uptake, consistent with the literature. Kurttio and Savolainen (1990) reported that personal ETU air concentrations in EBDC applicators (*n* = 43) were associated with urinary ETU concentrations. A small occupational exposure study (*n* = 13) reported associations between dermal mancozeb exposure and urinary ETU concentrations (Colosio et al. 2002). A recent experimental dermal exposure study on ETU in humans reported that approximately 10% of the dose of ETU applied on skin was excreted in urine (Ekman et al. 2013).

The higher concentrations of urinary ETU in immigrant women, which were explained partly by immigrant women living closer to banana plantations and washing agricultural workers clothes more frequently, suggests an inequity in living conditions and household activities between immigrant women and Costa Rican–born women. This inequity may result in increased urinary ETU concentrations in immigrant women compared with Costa Rican–born women. Immigrant status may be a proxy for a broad range of additional socioeconomic and environmental determinants of exposure and health, because it remained significant after adjusting for the other factors. In future studies, immigrant status should be an important consideration because it may modify associations between pesticide exposures and health (Bellinger 2008).

Because there is hardly any published information on factors that influence urinary ETU concentrations during pregnancy in women living near agricultural fields, we used manual stepwise selection for 21 variables to decide what variables should be included in the multivariable model. This procedure inflates type 1 error because the final model results from multiple testing with the same data set, so the estimated *p*-values (and confidence intervals) may be too small. However, even after correcting for multiple-comparison testing using the rather conservative Bonferroni criterion (Bland and Altman 1995), which results in a corrected *p*-value for statistical significance of 0.05/21 = 0.002, the variables residential distance, washing agricultural work clothes, and occupation in agriculture during pregnancy still remain statistically significant.

ETU exposure in this population of pregnant women is worrisome; 72% of pregnant women had EDIs_chronic_ above the RfD established by the U.S. EPA (1996). Also, pregnant women’s median urinary ETU concentrations were more than five times higher than those reported for other general populations (Aprea et al. 1996; Castorina et al. 2010; Colosio et al. 2006; Jones et al. 2010; Saieva et al. 2004), and comparable with post-shift urinary ETU concentrations of Italian agricultural workers (Colosio et al. 2002; Sottani et al. 2003) (see Supplemental Material, Figure S2). On occasion, women’s EDI_acute_ may reach the aPAD established by the U.S. EPA (2005).

To our knowledge, this is the first study to evaluate pesticide exposure metabolites in pregnant women living near agricultural fields with aerial spraying. In Costa Rica, pilots must maintain a distance of 100 m from residential areas in absence of a natural vegetative barrier, such as trees, and 30 m in presence of a natural vegetative barrier (LA GACETA 2008). Airplanes are equipped with geographical information systems to increase pesticide application precision and reduce off-target spray drift. Nevertheless, aerial applications have been perceived as hazardous by populations living near agricultural fields, and spraying distances are sometimes not respected (Barraza et al. 2011, 2013). In other countries, such as the United States, aerial pesticide applications have been associated with cases of off-target pesticide drift and acute pesticide illnesses (Lee et al. 2011).

In summary, pregnant women living near banana plantations had elevated urinary ETU concentrations compared with concentrations reported in previous studies, and their estimated EDI’s for chronic exposures often exceeded RfDs. Our findings suggest that current regulations governing aerial pesticide spraying activities do not protect pregnant women and fetuses from exposure to ETU, and that the principal source of exposure is likely to be aerial spraying of mancozeb. The factors predicting urinary ETU provide insight into possibilities for exposure reduction. Because of the inverse association of ETU with residential distance, the following measures would likely decrease both environmental and occupational exposures: reduction of aerial pesticide application frequency, replacement of aerial spraying with less dispersive application techniques, and implementation of additional technical measures to reduce spray drift. To reduce contamination of home environment, at minimum, the distance between banana plantations and residential areas should be increased, natural vegetative barriers should be planted, and work clothes should be washed not in the homes but at the workplace, using automated systems to avoid additional workers’ exposure.

## Supplemental Material

(322 KB) PDF
