# Erratum: “Aerial Application of Mancozeb and Urinary Ethylene Thiourea (ETU) Concentrations among Pregnant Women in Costa Rica: The Infants’ Environmental Health Study (ISA)”

**DOI:** 10.1289/ehp.122-A321

**Published:** 2014-12-01

In the Advance Publication of “Aerial Application of Mancozeb and Urinary Ethylene Thiourea (ETU) Concentrations among Pregnant Women in Costa Rica: The Infants’ Environmental Health Study (ISA)” by van Wendel de Joode et al. [Environ Health Perspect 122:1323–1328 (2014); http://dx.doi.org/10.1289/ehp.1307679], the unit given for daily creatinine release in pregnant women (1.21 g/day) should have been 1.21 g. It has been corrected in the final version.

The authors regret the error.

