# Supplementary material for: Aerial Application of Mancozeb and Urinary Ethylene Thiourea (ETU) Concentrations among Pregnant Women in Costa Rica: The Infants’ Environmental Health Study (ISA)
Source: Environ Health Perspect. 2014 Sep 8;122(12):1321–8. doi: 10.1289/ehp.1307679 (PMC4256696; doi:10.1289/ehp.1307679)
Supplement: (322 KB) PDF [file ehp.1307679.s001.508.pdf]

Supplemental Material

Aerial Application of Mancozeb and Urinary Ethylene Thiourea (ETU) Concentrations among Pregnant Women in Costa Rica: The Infants' Environmental Health Study (ISA)

Berna van Wendel de Joode, Ana María Mora, Leonel Córdoba, Juan Camilo Cano, Rosario Quesada, Moosa Faniband, Catharina Wesseling, Clemens Ruepert, Mattias Öberg, Brenda Eskenazi, Donna Mergler, and Christian H. Lindh

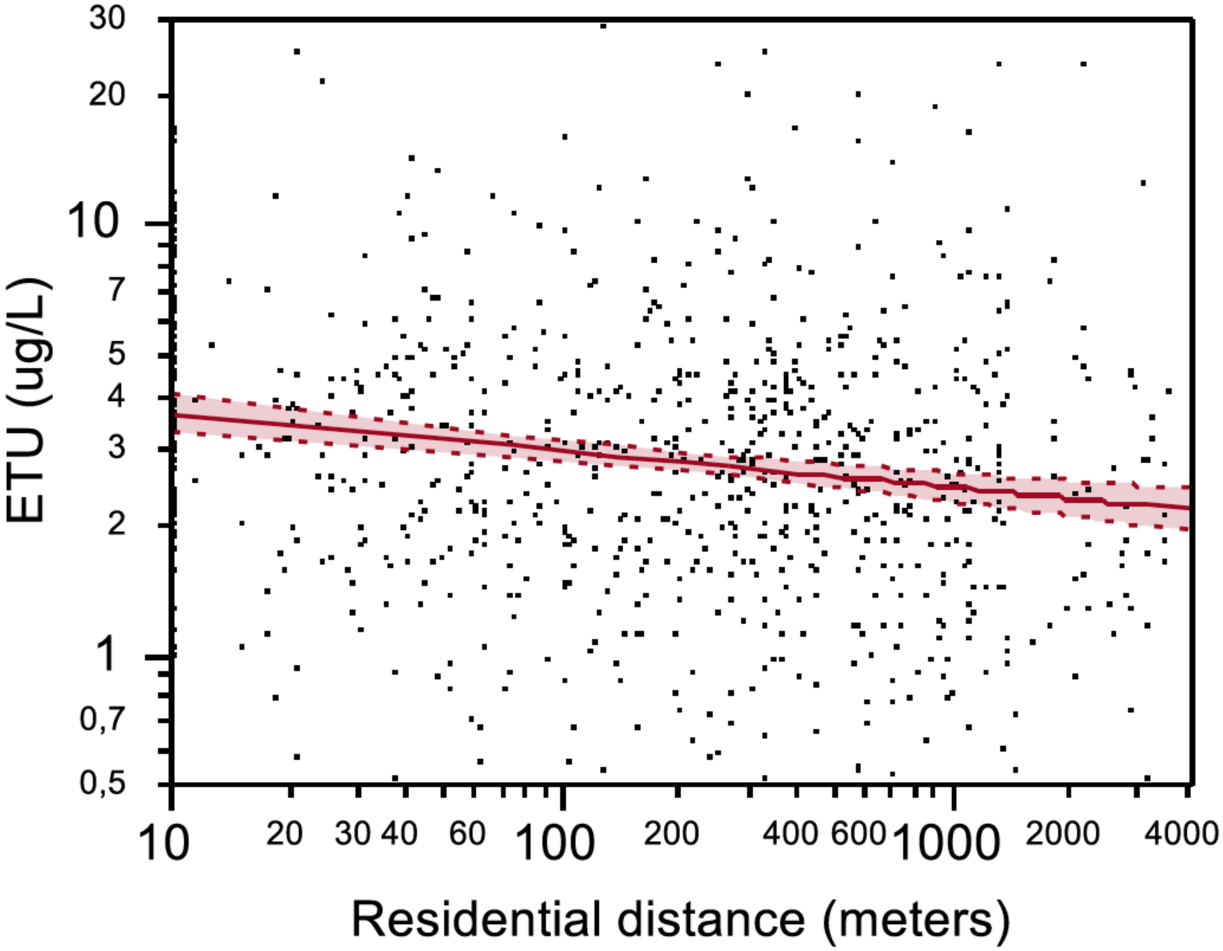

Figure S1. Association between residential proximity to banana plantation (ln(meters)) as independent variable and ln ETUconcentration as dependent variable using a multivariate mixed-effect model with woman included as a mixed effect and creatinine concentration (μ/L), occupation in agriculture during pregnancy, washed agricultural work clothes on day before sampling, immigrant as fixed effects; a -0.08% (95% CI -0.11, -0.05) decrease in urinary ETU for each 1% increase in distance (meters) ($p<0.0001$). Dashed lines represent 95% CI.

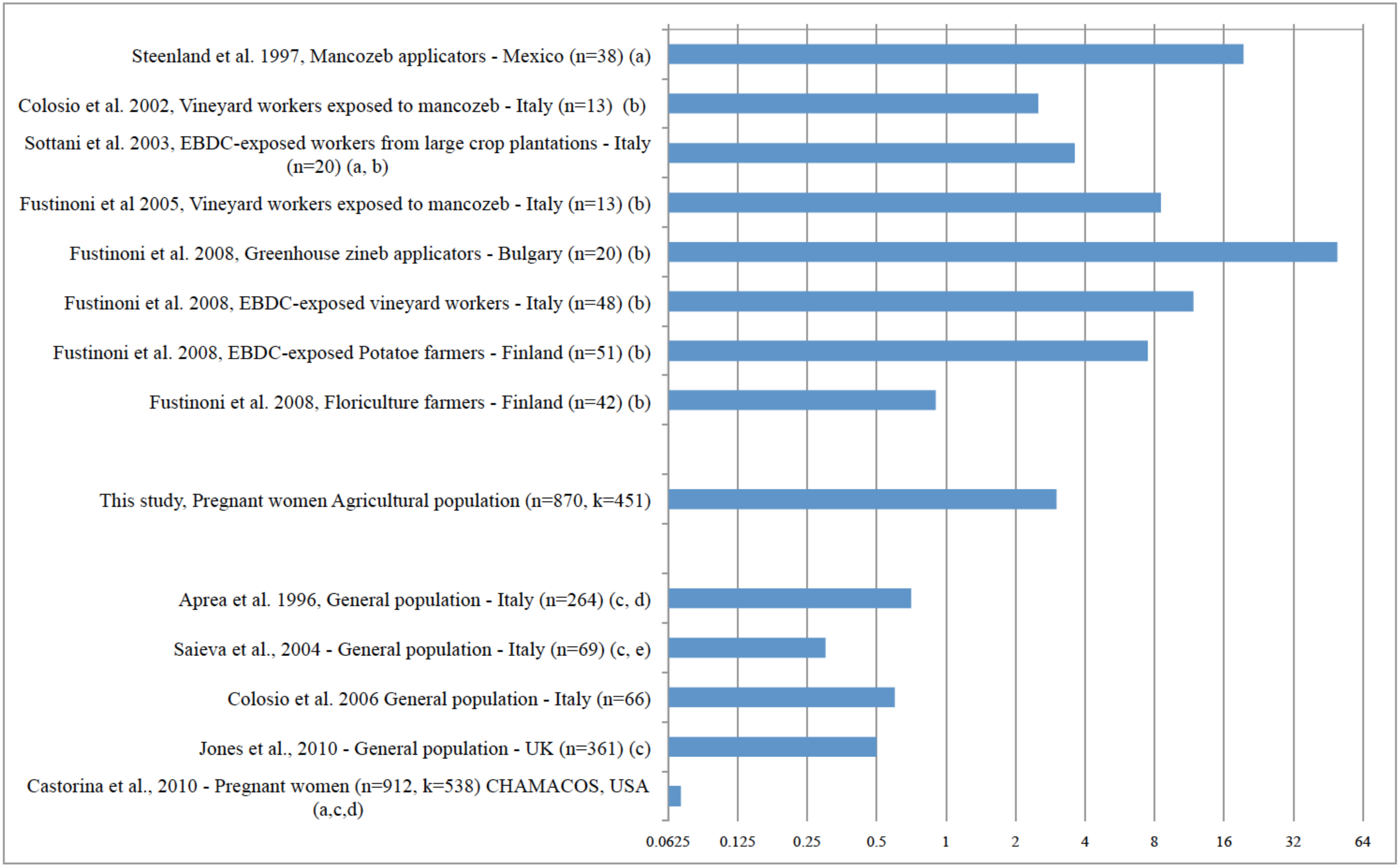

Figure S2. Geometric mean urinary ETU concentrations (µg/g.cr) in occupational (post-shift samples) and general populations as reported in literature. [a]Expressed in µg ETU/L urine. [b]Median value is presented, [c]>50% below LOD. [d]Since >75% of samples had concentrations below LOD, set at ½√2·LOD. [e]Conversion from nmol/day, assuming 1.6 L urine and 1.36 g creatinine/L (Jones et al. 2010).
